# Smart sharks: a review of chondrichthyan cognition

**DOI:** 10.1007/s10071-022-01708-3

**Published:** 2022-11-17

**Authors:** Culum Brown, Vera Schluessel

**Affiliations:** 1 School of Natural Sciences, Macquarie University, Sydney, Australia; 2 Institute of Zoology, University of Bonn, Meckenheimer Allee 169, 53115 Bonn, Germany

**Keywords:** Shark, Ray, Intelligence, Smart

## Abstract

450 million years of evolution have given chondrichthyans (sharks, rays and allies) ample time to adapt perfectly to their respective everyday life challenges and cognitive abilities have played an important part in that process. The diversity of niches that sharks and rays occupy corresponds to matching diversity in brains and behaviour, but we have only scratched the surface in terms of investigating cognition in this important group of animals. The handful of species that have been cognitively assessed in some detail over the last decade have provided enough data to safely conclude that sharks and rays are cognitively on par with most other vertebrates, including mammals and birds. Experiments in the lab as well as in the wild pose their own unique challenges, mainly due to the handling and maintenance of these animals as well as controlling environmental conditions and elimination of confounding factors. Nonetheless, significant advancements have been obtained in the fields of spatial and social cognition, discrimination learning, memory retention as well as several others. Most studies have focused on behaviour and the underlying neural substrates involved in cognitive information processing are still largely unknown. Our understanding of shark cognition has multiple practical benefits for welfare and conservation management but there are obvious gaps in our knowledge. Like most marine animals, sharks and rays face multiple threats. The effects of climate change, pollution and resulting ecosystem changes on the cognitive abilities of sharks and stingrays remain poorly investigated and we can only speculate what the likely impacts might be based on research on bony fishes. Lastly, sharks still suffer from their bad reputation as mindless killers and are heavily targeted by commercial fishing operations for their fins. This public relations issue clouds people’s expectations of shark intelligence and is a serious impediment to their conservation. In the light of the fascinating results presented here, it seems obvious that the general perception of sharks and rays as well as their status as sentient, cognitive animals, needs to be urgently revisited.

## Introduction

Sharks and their relatives have inhabited the oceans for more than 400 million years. Currently, there are around 1200 extant species that occupy almost any aquatic environment. Their morphologies and behaviours are as diverse as their ecologies and lifestyles. The smallest ray is only about 10 cm long (electric rays from the Narcinidae), whereas a whale shark (*Rhincodon typus*) can reach 10 m or more in length. Some are apex predators while others are planktivores. While many of us immediately picture top pelagic predators such as the white shark (*Carcharodon carcharias*) when we think of sharks, in fact most species are benthic. Rays make up more than 50% of the diversity. The large breadth of variation observed in all aspects of elasmobranch (sharks and rays) biology is expected to be reflected in a large variation in brains and behaviour (Yopak 2012a, b), but the study of cognition in sharks and their relatives is still very much in its infancy, largely due to the many impracticalities of working with large aquatic animals (Schluessel 2015). Because of these limitations, most studies have been conducted on small benthic species that are easy to handle or maintain in captivity, such as Port Jackson sharks (*Heterodontus portusjacksoni*), bamboo sharks (*Chiloscyllium griseum*) and some of the smaller ray species (see review Guttridge et al. 2018). The only large, benthopelagic species assessed in any detail is the lemon shark (*Negaprion brevirostris*) thanks largely due to work at the Bimini Field Station in the Bahamas.

**Fig. 1:**
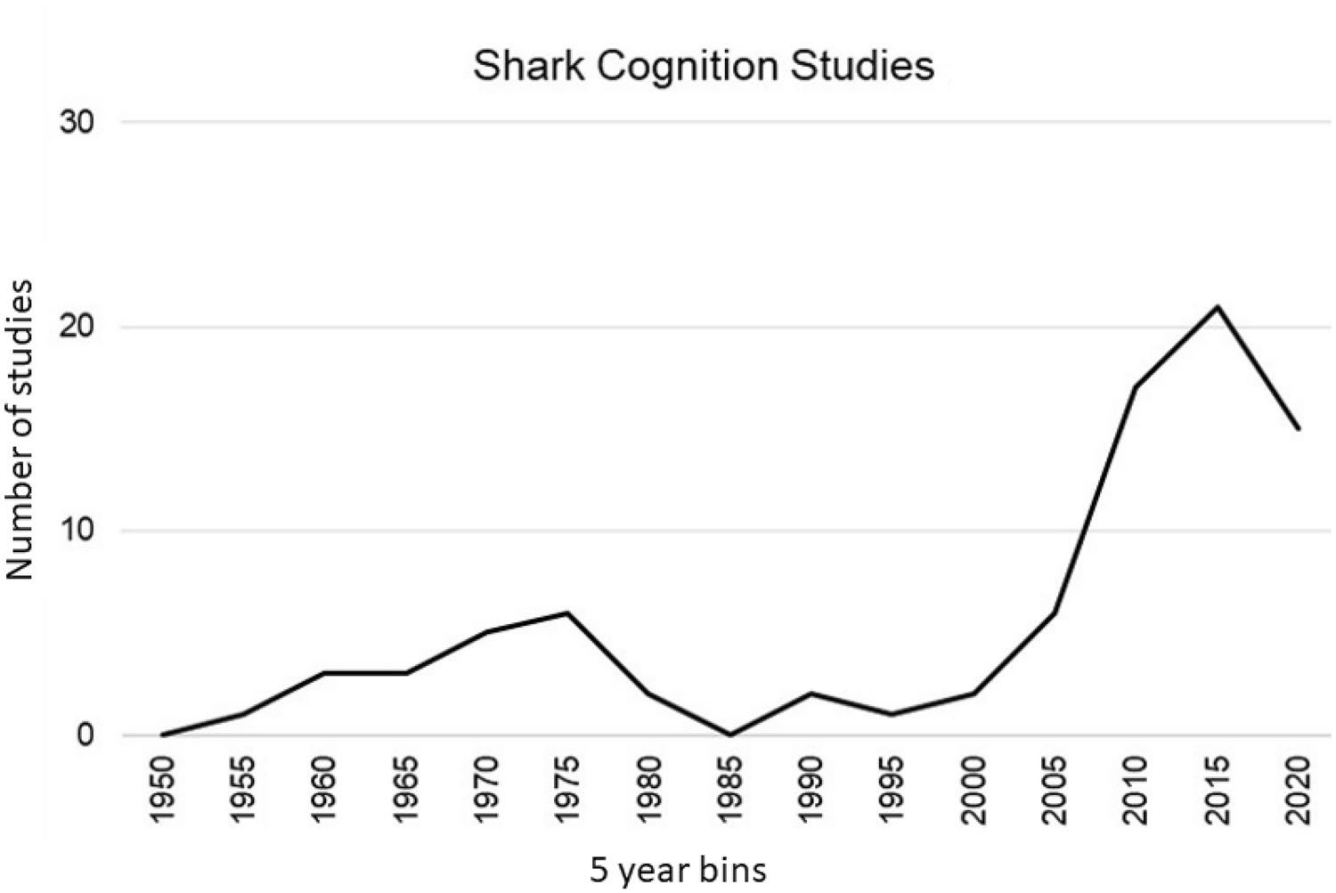
Increase in the number of published papers examining cognition in sharks and rays (presented in 5-year intervals. Note that studies depicted in 2020 are only from a 2.5-year interval)

Shark cognition has been reviewed several times in the past ten years (Guttridge et al. 2009, 2018; Schluessel 2015), but in the last few years there has been a huge increase in the number of studies examining shark cognition (Fig. 1). While traditionally the study of shark cognition has occurred under captive or semi-captive conditions, there is an emerging trend to study sharks in the wild, largely facilitated by improvements in technology such as animal tagging (Hussey et al. 2015). While it may be tempting to assume that the cognitive ability of sharks and rays may be similar to teleost fishes because they inhabit similar ecosystems, the reality is we have studied far too few sharks and rays to determine the impacts of ecology on cognition in this taxa. However, it does appear that sharks and rays have a similar cognitive toolbox to the rest of the vertebrates (Guttridge et al. 2018). This is perhaps not surprising, given that the fundamental structure of the vertebrate brain is highly conserved (Yopak 2012a, b). Here, we review papers on chondrichthyan cognition concentrating on these newer studies while referring to earlier studies to provide context. For an excellent historical perspective of learning and memory in sharks see Guttridge et al. (2009).

## Auditory stimuli

Sound travels remarkably well under water and likely plays an important role in the lives of aquatic animals but can easily be disrupted by human activities (Kunc et al. 2016). Sound discrimination is very well studied in birds and humans (Burgering et al. 2018) but less so in sharks and rays. Fishermen and cage diving tourism operators claim that sharks learn to recognize the sound of their boat engines and approach. Indeed, depredation on both recreational and commercial fish catches strongly suggests that sharks associate fishing vessels with a free meal (Mitchell et al. 2018a). In Exmouth, Western Australia for example, Mitchell et al. (2018b) found that nearly 40% of all recreational fishing trips reported depredation incidents. Depredation tends to be higher in locations where fishing pressure is highest, suggesting sharks are either attracted to the consistent cues associated with fishing activity (smell of fish blood, boat engine noise, etc.) and/or learn to stay in these locations via conditioning. Acoustic tracking of bull sharks (*Carcharhinus leucas*) in South Africa showed that they spent considerable time close to fishing boats and made clear movements towards fishing boats in response to engine noise and hauling the anchor chain (McCord and Lamberth 2009). These anecdotal lines of evidence suggest that sharks can associate artificial sounds with food rewards. Vila-Pouca and Brown (2018) examined this concept in the laboratory by pairing a food reward with an artificial sound (jazz music). Juvenile Port Jackson sharks had to swim to a specific zone located within a larger arena when jazz was played to get a food reward. Five out of eight sharks learnt the task. But when asked to discriminate between jazz and blues the sharks they failed. Sharks are, however, capable of discriminating between different sounds. Grey bamboo sharks (*Chiloscyllium griseum*), for instance, differentiated between two low frequency sounds during a Go/No-Go procedure (Poppelier et al. 2022). Sensory cues appear to be of varying importance to bamboo sharks when performing simple discrimination tasks. While both acoustic and visual cues are perceived and memorized, visual cues seem to be more important when placed into conflict with acoustic cues, indicating that at least in this context, vision is the more dominant sensory system (Poppelier et al. 2022; Halbe 2018). Similar findings have been found in elephants which preferentially use smell rather than sound to locate food rewards (Plotnik et al. 2014).

## Visual stimuli

The visual system is probably the most extensively researched sensory system in elasmobranchs, both in general and in the context of cognition studies. An early study by Kuba et al. (2010) showed that stingrays (*Potamotrygon castexi*) rapidly learned to use water as a tool to extract food from a tube presented with a positive (rewarded side) and a negative (blocked side) stimulus indicated by black or white tape. Daniel and Schluessel (2020) conducted a serial reversal experiment on the ocellate river stingray (*Potamotrygon motoro*) using two-dimensional geometric objects in a visual two-alternative forced-choice task. Reversal tasks are often used as indicators for cognitive flexibility. Five of seven stingrays reversed successfully at least once, but all of them required significantly more sessions for the first reversal than during training. Performance was highly variable, with only one stingray demonstrating progressive improvement across all four reversal phases. In contrast, bamboo sharks that were trained in the same serial reversal procedure (Leo 2019; Schluessel unpublished data) halved session times during the first reversal relative to training and further reduced session number to reach criterion over the course of up to ten reversals. Dogs trained to discriminate between different plates were also marginally quicker to learn the reversal task compared to training (Piotti et al. 2018). These data show that bamboo sharks can inhibit a previously learned association more quickly with experience, perhaps by developing strategies for rule identification.

Recent visual discrimination experiments in stingrays (*Potamotrygon motoro*) showed that stingrays, like sharks (Fuss et al. 2014a), are able to discriminate stimulus presence and absence, overall stimulus contrasts, different forms, horizontal from vertical stimulus orientation, as well as different colours that also vary in brightness (Daniel et al. 2021). As opposed to all previously assessed shark species, which are monochromats and therefore colour-blind (Hart et al. 2011; Schluessel et al. 2015), ocellate river stingrays possess two different cone types (*lws*, *rh2*) and can differentiate colours as part of a visual colour system that is ecologically adapted to a riverine habitat. Colour vision independent of brightness was further tested by training stingrays to differentiate between red and green plates, as well as blue and yellow plates (Schluessel et al. 2021). Red hues of different brightness were also distinguished from one another to a degree significantly above chance level (Schluessel et al. 2021). Moreover, stingrays tested in simple visual resolution experiments demonstrated a range of visual acuities from < 0.13 to 0.23 cpd (Daniel et al. 2021). This study also provided the first evidence for memory retention in discrimination learning in this species, while memory retention in sharks (Fuss and Schluessel 2015) and spatial memory abilities in stingrays and sharks had already been examined (Schluessel and Bleckmann 2005, 2012).

**Fig. 2:**
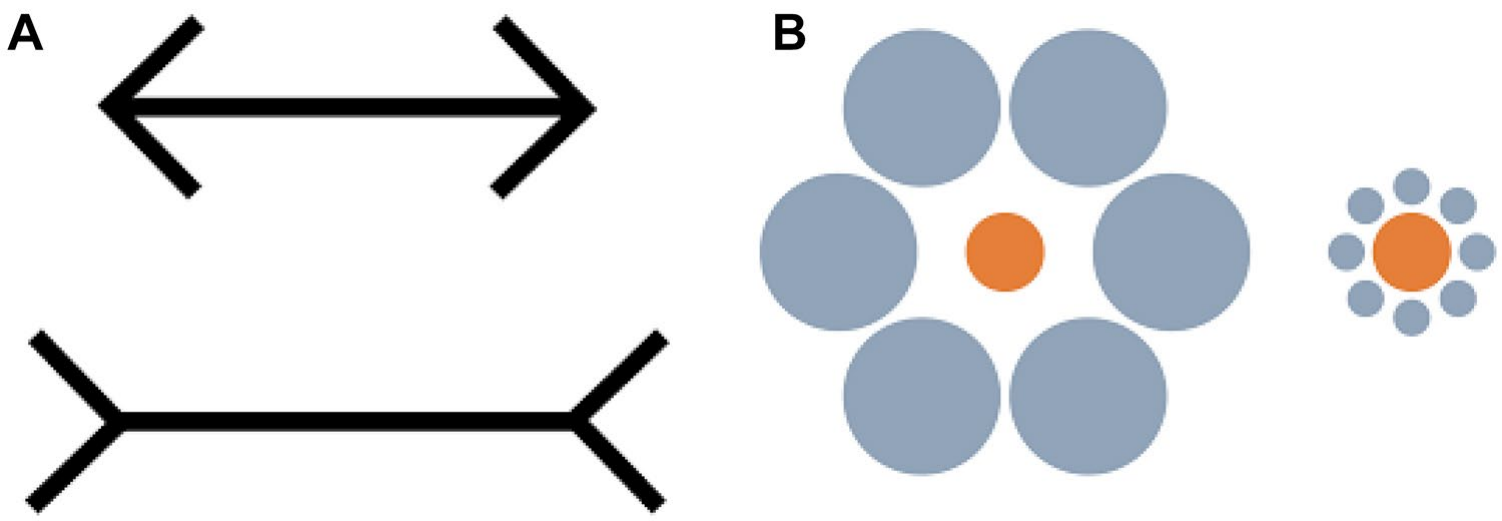
Sharks are not fooled by Müller-Lyer illusion (**A**) and Ebbinghaus-Titchener circles (**B**)

Fuss et al. (2014b) found that bamboo sharks, just like humans, succumb to optical illusions including the Kanizsa figures or subjective contours but not to the Müller-Lyer illusion (Fig. 2). A follow up study (Fuss and Schluessel 2017) showed that bamboo sharks are also not fooled by Ebbinghaus-Titchener circles and variations of the Delboeuf illusion. Unlike humans, dolphins, several bird species or damselfish, they ignored the illusory effect and preferred the overall larger image. The effect of learning and differentiating visually between geometric and illusory contours was also investigated neuranatomically. A significant up-regulation of the immediate early gene (IEG) egr-1 expression levels in a small region of the caudal telencephalon was revealed in all trained sharks compared to controls (Fuss and Schluessel 2018). This region seems to be involved in information processing and or higher visual functions. The results suggest that there are important similarities between elasmobranchs and other vertebrate groups in the neuronal plasticity and activity-dependent IEG expression in the brain.

Categorization, a process whereby objects are grouped together according to some defining feature, is a central process of cognition (Harnad 2017). It has been studied in sharks using basic visual categories (two-dimensional objects of fish and snails, Schluessel and Duengen 2015) as well as the more abstract concept of symmetry vs. asymmetry (Schluessel et al. 2014a; b). Fuss et al. (2021) extended this work by studying abstract relation concepts in greater detail using more complex tasks. Bamboo sharks had to distinguish different pairs of abstract, geometric objects that satisfied two conceptual preconditions, i.e. same vs different, as well as a particular spatial arrangement (horizontally/vertically arranged). While bamboo sharks learned both rules, they primarily chose objects based on ‘difference’ and also applied this rule in transfer tests where they had to choose between unfamiliar objects that had not been used during the association process.

**Fig. 3:**
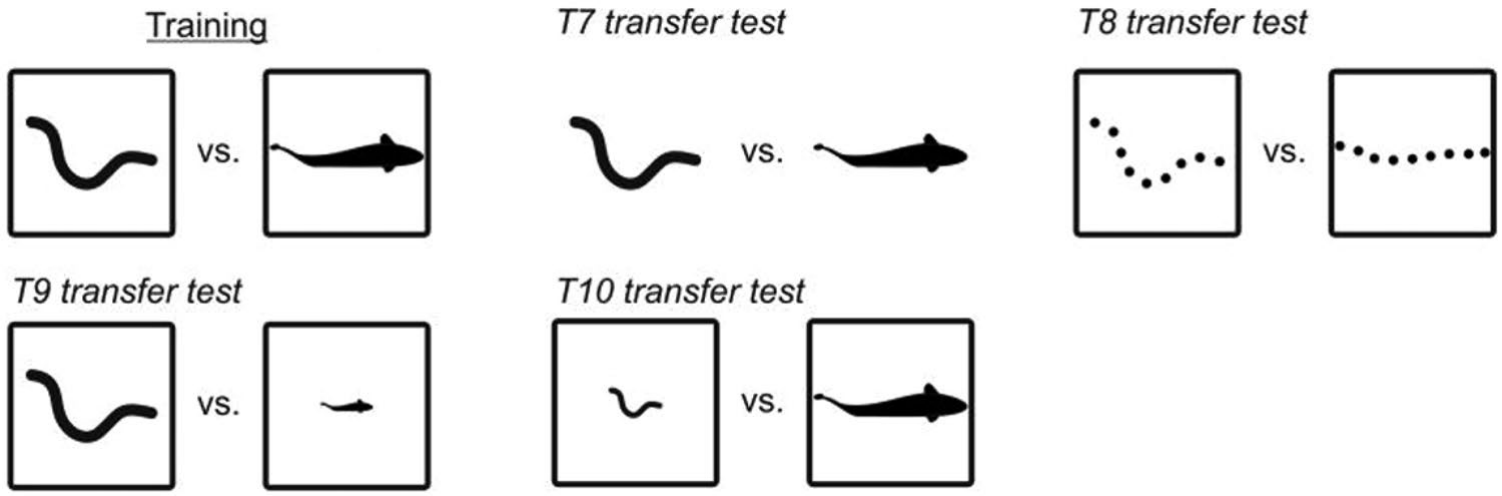
Grey bamboo sharks can use point displays to recognise and differentiate between different forms of biological motion and recognize a familiar organism solely based on its movement pattern (after Fuss et al. 2017)

The aquatic environment is highly dynamic, things are rarely static. Detection of moving stimuli is a research field of particular interest in this context but has not received much attention in elasmobranchs. Fuss et al. (2017) tested if juvenile grey bamboo sharks could visually perceive and discriminate simple and complex motion patterns using a two-alternative forced choice paradigm. Sharks were first successfully trained to differentiate between videos featuring two circles moving at a range of different velocities. Subsequent transfer testing revealed that the training stimulus was still successfully detected if velocity or direction of the alternative stimulus changed. In a second task, individuals were presented with more complex motion patterns in form of videos of different organisms such as an eel vs. a trout, an eagle vs. a bat or a dolphin vs. a shark. A series of transfer tests elucidated whether sharks could still recognize these stimuli when shown from different perspectives (front or sideways), when enlarged or downsized, or when presented in form of a point display (PDs). Point displays or point light displays (PLDs) are commonly used to depict the concept of biological motion. Johannson (1973) discovered that humans immediately recognize different forms of human movement even when these patterns are abstracted and presented without any figural information, i.e. when the presentation of the organism is reduced to the movement itself. To study this, light dots were placed on specific joint positions along a moving human body dressed in black while being presented against a black background, creating PLDs (PDs are the opposite, black circles on a light background). Ensuing studies found that humans can also infer information on gender (Kozlowski and Cutting 1977), emotions (Dittrich et al. 1996) and recognise friends and family members (Cutting and Kozlowski 1977) from PLDs. Results for sharks were rather surprising, as they easily discriminated between differently moving organisms but failed in two out of three experiments to apply the acquired information to new situations in transfer tests. In contrast, cichlids trained in the same procedure performed much better (Schluessel et al. 2015, 2018). Nonetheless, in the third experiment, sharks differentiated between the PDs of an eel and a trout, suggesting that the ability to spontaneously recognize an organism based on movement alone is present in elasmobranchs (Fig. 3).

## Numerosity

Numerosity has been studied in a wide range of animals and results suggest that the mechanisms used seem to be universal (Agrillo 2015). Visual discrimination experiments have been used to study numerical abilities in sharks and rays. Being able to differentiate quantities (‘more’ or ‘less’) or even exact amounts is vital for animals in the context of foraging, predator avoidance, schooling and mate choice. Quantity discrimination can be based on several parameters such as continuous variables (e.g. size or surface area covered by items) or discrete variables (number of items). While these strategies are not mutually exclusive and may, when combined, yield a more successful outcome, some species use only continuous variables such as size to base their choice on and seem unable to use numerical information by itself (Agrillo et al. 2011). Quantity discrimination abilities in elasmobranchs were first demonstrated in Port Jackson sharks (Vila Pouca et al. 2019). While freshwater stingrays and bamboo sharks were found to use numerical information independently of other variables, the maths did not come easy to them—only about half the animals tested learned the training task and went on to perform in transfer tests. The upper limit, up to which two amounts of presented objects differing by only one item could still be successfully differentiated, was four (Kreuter et al. 2021), which is similar to most vertebrates (Agrillo 2015). A follow up study (Schluessel et al. 2022) tested the stingray’s addition and subtraction abilities within the number space of one to five. Based on a protocol established for honeybees (Howard et al. 2019), stingrays learned to perform either an addition or subtraction, depending on the presentation of two-dimensional objects in two distinct colours, with the colour signalling a particular arithmetic process. Only half of the stingrays successfully completed training and recognized specific colours as symbols for addition and subtraction. Nonetheless, those that passed the initial test needed fewer sessions than cichlids to reach criterion and excelled in both training and transfer tests. Subjects also learned to specifically add and subtract the factor ‘one’ instead of simply learning the rule to always pick the highest or lowest number presented. The ability to add and subtract is consistent with the idea that many animals use an ‘object tracking system’, i.e. Object File System (OFS) when operating within this number range (Feigenson et al. 2004; Agrillo et al. 2015; Dadda et al. 2015).

## Electrical and magnetic cues

Sharks are very sensitive to electrical and magnetic cues which conduct well through water (Schluessel 2020). Anderson et al. (2017) conditioned juvenile sandbar sharks to a magnetic stimulus to which they reacted strongly by increasing tail beat frequency and swimming speed. Response rates were 100% to all magnetic field intensities tested, ranging from 0.03 to 2.89 µT above the ambient geomagnetic field. In another behavioural study, yellow stingrays (*Urobatis jamaicensis*) were trained to associate a magnetic stimulus with a food reward in order to elicit foraging behaviour (Newton and Kajiura 2017). In the absence of any other sensory cues, magnetic (about 100 mT) and non-magnetic stimuli were hidden at random locations within a test arena and rays were successfully conditioned to discriminate between them. For comparison, a study examining the detection of magnetic anomalies in pigeons in a conditioned choice experiment used 145 µT above background levels (Mora et al. 2004). In a follow up study by Newton and Kajiura (2020a), yellow stingrays were trained to use the polarity of the geomagnetic field (i.e. the north–south direction) as an orientation cue to find food in a T-maze. All stingrays reached the learning criterion and were then reverse trained to associate the previously unrewarded magnetic stimulus (i.e. featuring the opposite polarity) with the reward location. Interestingly, the stingrays reached the learning criterion in the reversal task significantly faster than in the initial procedure. A third study (Newton and Kajiura 2020b) tested whether yellow stingrays could detect and distinguish between geomagnetic field (GMF) cues to determine their location. They found that yellow stingrays could distinguish between changes in GMF intensity and inclination angle and may use this information to establish a magnetically based cognitive map. It is well established that other animals, such as marine turtles or birds, use magnetoreception for navigation (Mouritsen and Ritz 2005; Lohmann 2010; Putman et al. 2011).

## Chemical cues

It is perhaps unsurprising that sharks and rays can make associations with chemical cues, but experience early in life can often have lasting impacts on behaviour. Studies suggest that exposure to the smell of predators as embryos shapes the behavioural response to predator cues upon hatching (Ferrari and Chivers 2009). Rainbowfish embryos, for example, were exposed to chemical cues emanating from a novel predator, a native predator and injured conspecifics at just four days post fertilisation (Oulton et al. 2013). The embryos’ response was measured by changes in heart rate, which revealed the strongest response to the smell of a native predator. These data indicate that the nervous system is laid down very early in development and begins functioning well before hatching. Similar studies were conducted on Port Jackson shark embryos at different developmental stages to determine if they could differentiate between the smell of fish or sharks. Rather than observing heart rate, Gervais et al. (2021) measured changes in oxygen consumption as a proxy for metabolic rate to record their responses: freeze (depressed oxygen consumption) or flight (enhanced oxygen consumption). Embryos at stage 13 and 14, shortly after the egg mucous plug dissolves, suppressed metabolic rate (a freeze response) when exposed to the smell of a teleost but increased their metabolic rate in response to the smell of a horn shark (flight response) at stage 15 (hatching). Thus, shark embryos seem to be able to differentiate between chemical cues emanating from different sources and show appropriate responses particularly in the latter stages of development.

Learning to pair the smell of food with various environmental cues has also been studied in wild juvenile Port Jackson sharks, which were captured and transported to an aquarium. Adjacent to the housing area, a conditioning experiment was set up where the sharks had to learn to associate a burst of bubbles or a light with a food reward. The sharks learnt both associations in less than 10 days. Memory retention tests suggested they remembered the association for up to 40 days post training illustrating a remarkable capacity for long term memory (Guttridge and Brown 2014).

The smell of food is often used to lure in sharks and rays in ecotourism operations. A tantalising outcome of the capacity for sharks to learn associations with human behaviour and or infrastructure is how best to manage issues that may result in conflict. Cage dive operators often attract sharks to the cage to enhance the experience of their customers. In many states and countries, it is illegal to attract sharks using food because of a fear of substantially altering the behaviour of the shark over the long term, and operators often use the smell of food as an alternative (Gallagher and Huveneers 2018). This raises the question as to what reinforcement regime would entice the shark enough to interact with cage divers, but not so much to cause dependency on the provisioned food. There is a delicate balance to be struck. Heinrich et al. (2020a, b) used an operant conditioning task to determine the impacts of reinforcement frequency and reward magnitude on memory retention in Port Jackson sharks. Those trained at high reinforcement frequency learnt the task faster but the size of the reward had little influence. This suggests that altering the frequency of reinforcement might be the most effective management strategy. Collectively these tests suggests that sharks can rapidly make associations with a range of natural and artificial cues. Given that human activities are increasingly prevalent in the marine environment we expect that sharks and rays readily use these cues if they predict important events.

## Social cognition

Understanding the social behaviour of sharks and rays has important implications for conservation biology (Jacoby et al. 2011). We have established that adult Port Jackson sharks like to spend time with particular individuals using social network analysis of acoustic tracking data (Mourier et al. 2017a) and are frequently observed stacked on top of one another in crevices and caves during the day (pers. obs). But casual observations suggested that juvenile Port Jackson sharks appear to be more solitary. By conducting simple sociality tests where subjects got to choose between various contrasting social contexts located at either end of an aquarium, Villa-Pouca and Brown (2019) wanted to establish if juveniles showed any preferences for conspecifics. They found that baby Port Jackson sharks spent time close to food when it was in the stimulus area, but they did not seem to avoid or approach unfamiliar conspecifics and, if anything, showed avoidance of familiar conspecifics in the first five minutes of the trial. This contrasts with previous studies on spotted catsharks and lemon sharks, where familiarity seems to be an important social factor (Jacoby et al. 2012; Keller et al. 2017), suggesting that captive juvenile Port Jackson sharks are slightly anti-social. But despite this, they are capable of learning by watching or interacting with others (social learning; Brown and Laland 2003). Sharks were trained to solve a simple binomial choice test where they had to swim through the correct door to access a food reward. Afterwards, naïve sharks were allowed to either interact with these trained demonstrators or with sham demonstrators. Those sharks trained with demonstrators where far more likely to learn the task than those trained with sham demonstrators (Vila Pouca et al. 2020). Similar observations of social learning have been made in stingrays (Thonhauser et al. 2013).

**Fig. 4:**
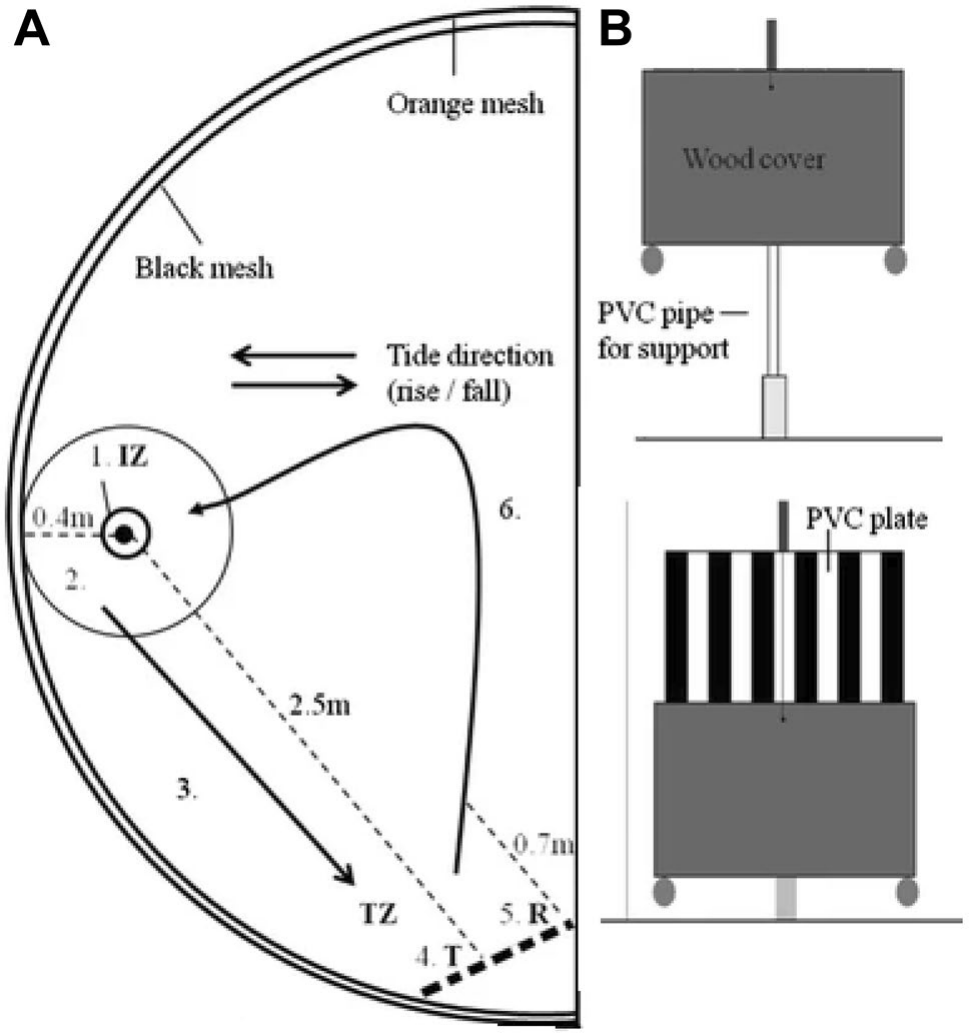
Social learning task for lemon sharks (after Guttridge et al. 2013) in a semi-captive condition (**A**). Sharks had to enter the indicator zone (IZ) which caused a target (T) to be revealed (**B**). Subjects then had to approach the target and enter the target zone (TZ) to receive a food reward (R)

Guttridge et al. (2013) studied social learning in sharks under semi-captive conditions. Wild lemon sharks (*Negaprion brevirostris*) were captured and transferred into pens in the shallow waters of Bimini to settle prior to experimentation. Initially demonstrator sharks were trained to complete the foraging task and then paired with naive sharks to see how quickly the naïve sharks would learn the task. In the control situation, naïve sharks were paired with sham demonstrators that had no prior experience. The task was reasonably complex (Fig. 4): Subjects had to move into the indicator zone (IZ) which revealed a target (T) on the other side of the enclosure. They then had to approach the target to receive a food reward. The demonstrator pairs out-performed the sham pair during the training phase as indicated by the median duration to complete 5 trails (1 session). Then, in the test phase when the demonstrators were removed, observers that had been paired with demonstrators continued to perform well having significantly more visitations to the indicator and target zones compared to sharks trained with sham demonstrators. Papastmatio et al. (2022) speculated that white sharks may use social contacts to share information about the location of large prey. These results suggest that sharks and rays frequently rely on socially acquired information to solve novel problems. Studies in teleosts have found that individuals balance public and private information when making decisions (Trompf and Brown 2014), but no such studies have been conducted in elasmobranchs.

Acoustic tracking data enables scientists to establish who is spending time with whom and thereby probe the social intelligence of sharks in wild populations (Papastamatiou et al. 2022a, b). In Port Jackson sharks, for example, we found that the social relationships formed in breeding aggregation sites in Jervis Bay (Australia) are consistent from year to year and Port Jackson sharks choose who they spend time with based on sex and by size but not relatedness (Bass et al. unpublished data). Similar observations have been made in reef sharks (Papastamatiou et al. 2020; Mourier and Planes 2021). Importantly, shark social networks are often highly structured, meaning their association patterns are not random (Mourier et al. 2017a; Anderson et al. 2021). Intriguingly, the social relationships between Port Jackson sharks at breeding aggregation sites not only determine the time at which they depart on their southerly migration but also predict when they will arrive the following season (Pelizza et al. unpublished data). Social network analysis of a manta ray population in West Papua based on individual identification using unique spots as well as acoustic tags has also shown that the social relationships between these animals is far more complex than previously believed (Perryman et al. 2019, 2022). Using a similar approach for black tip reef sharks in French Polynesia, Mourier et al. (2017b) interrogated the social network to see how robust it was to fishing pressure. The study found that in a pristine environment there is considerably redundancy in network affiliations and the network only began to fragment under considerable fishing pressure. Interestingly, sharks learned to avoid being recaptured during this study. Learned hook avoidance is quite common in fishes (Lovén Wallerius et al. 2020).

Based on observations of visually tagged rays competing for access to fish discards, Pini-Fitzsimmons et al. (2021) used heterarchy, a combination of social network analysis and hierarchies, to investigate the social lives of smooth rays. They found that their interactions when competing over food scraps were indicative of a despotic society centred on a big female called Raylene. These complex social systems are typically found in primates and other highly social mammals (King et al. 2008). Using a similar tiered analytical approach, Jacoby et al. (2021) found subtle impacts of marine tourism provisioning on the social behaviour of tiger sharks (*Galeocerdo cuvier*) in the Bahamas. Bull sharks (*Carcharhinus leucas*) are often considered solitary but provisioning at a dive site in Fiji facilitated the development of social associations (Bouveroux et al. 2021). Collectively, the work on the social lives of sharks and rays has shown that they are far more sophisticated than previously believed. Many species show preferences for spending time with particular individuals based on size, sex and familiarity and these relationships can be stable over long periods of time. These studies also highlight the importance of redundancy in social networks and suggest that social relationships in wild populations are vulnerable to anthropogenic disturbances particularly if key individuals are removed from the network. Fragmentation of populations can lead to species decline and loss of cultural information transfer.

## Spatial learning

Spatial cognition is one of the best studied cognitive abilities in bony fish (Vila Pouca and Brown 2017) and is also well studied in elasmobranchs. Many of the experiments mentioned above have spatial elements to them. In the laboratory, studies on spatial memory and orientation strategies have mainly focused on freshwater stingrays and bamboo sharks. Experiments have shown that both species can use multiple orientation strategies (Schluessel and Bleckmann 2005, 2012), remember a spatial task for at least six weeks (Schluessel and Bleckmann 2012) and that allocentric strategies in sharks are processed in the telencephalon (Fuss et al. 2014c), while egocentric ones are not (Fuss et al. 2014d), which closely matches results for teleost fish and other vertebrates (Broglio et al. 2011). When studying the spatial learning ability of freshwater stingrays using a hole-board task, small alterations in the positioning of signalling landmarks caused individuals to visit both locations equally often (likely because of spatial conflict between the original feeding location and the new beacon positioning), whereas large alterations lead to an abandoning of the beacon in favour for choosing the original feeding location (Schluessel et al. 2015). In additional experiments, stingrays remembered the position of both proximate and distal landmarks as well as memorizing particular swimming paths. Results showed that rays generally placed more importance on the overall environmental or geometric arrangement (global cues) of the experimental arena than on individual landmarks (local cues), which may be ecologically more reliable. A further study on the use of two egocentric strategies, i.e. direction vs. landmark learning, found that stingrays utilize the former much more readily and frequently, even in different experimental set ups (Schluessel and Ober 2018). Comparative studies using different populations of fishes suggest that the use of different spatial learning cues is likely linked to the environmental conditions where the animals normally live (Odling-Smee and Braithwaite 2003).

Much of the research on chondrichthyan spatial cognition in the field involves tagging sharks with acoustic transmitters. The acoustic tags give off a series of pings that enable them to be identified by a receiver. The receiver may be stationary in the case of passive tracking, or hand-held / boat mounted in the case of active tracking. By tracking where sharks are and when, one can find out all sorts of things about shark behaviour and provide insights into their cognition. For example, studies on lemon sharks in Bimini found that sharks returned home after being displaced by up to 16 km (Edren and Gruber 2005). Similarly, acoustic tracking data has revealed complex migration routes exhibited by Port Jackson sharks (Bass et al. 2016). On the east coast of Australia, Port Jackson sharks aggregate in coastal embayments to breed during the winter months. They migrate south into the cool waters of Bass Strait for summer and returns the following winter. Some individuals make return journeys well over 1000 km. The sharks not only return to the same bay, but to the same rocky reef within the bay year after year. Females occasionally skip a year, probably because they do not have sufficient energy to reproduce. These findings suggest that sharks have incredible navigation skills on par with the much-celebrated salmonids. How sharks navigate over such vast distances remains something of a mystery, but they likely rely on a wide range of cues including the Earth’s geomagnetic field (Klimley 1993; Schluessel 2020; Keller et al. 2021), olfaction (Nosal et al. 2016) and cognitive maps (Schluessel and Bleckmann 2005, 2012; Meyer et al. 2010) which is likely similar to migrating birds. Another intriguing question is how do juvenile sharks learn the routes on these vast navigational trips? There is no parental care in many species of sharks and rays, and the juveniles may spend some years in natal areas before dispersing. We hypothesise that migration routes may be passed on culturally, that is by social learning. Cultural inheritance of migration routes is likely common in many fish species (Brown 2022).

## Time-place learning

The natural world is often dictated by rhythms that enable animals to predict where important resources are likely to be in space and time. Field data on gulls, for example show that at sunrise they flew to specific locations to feed on earth worms, but only after rainfall; a form of conditional time-place learning (Wilkie et al. 1997). At the Woolamia boat ramp in Jervis Bay, Australia, smooth stingrays (*Dasyatis brevicaudata*) have been provisioned with fish scraps from fish cleaning facilities for at least 30 years. The rays have learnt to associate the behaviour of fishers with the arrival of food. Tagging and tracking data shows that the rays arrive at the boat ramp between 10 and 2 pm, particularly on weekends, corresponding to the peak time of fish cleaning activity (Pini-Fitzsimmons et al. 2018). Unlike the gulls, the rays even show up on rainy days when there are no fishers present, suggesting they are partially dependent on this artificial source of energy. This is a prime example of time-place learning in wild elasmobranchs responding to anthropocentric cues. Obviously, weekends are not a natural occurrence in the wild, but because human behaviour and rhythms are dictated by the day of the week, this has flow-on effects on wildlife behaviour.

Heinrich et al. (2020a, b) wanted to establish how quickly sharks and rays can learn about provisioning events that are predictable in time and space. To this end they established a provisioning station in the shallow lagoon in Bimini in the Bahamas and initiated feeding once per day for 27 days. Six lemon sharks were fitted with acoustic transmitters to monitor their movements. After 11 days of provisioning the sharks began to anticipate food by arriving at the provisioning location in the hour prior to the feeding event. Sharks retained anticipatory behaviour for up to 90 days after provisioning had ceased, which clearly shows that conditioning in this context is long lasting and that there are long-term implications for provisioning marine wildlife (Heinrich et al. 2020a, b).

## Chondrichthyan cognition in the Anthropocene

As we have seen in many examples above, wild sharks and rays respond to anthropogenic cues and like all marine animals, sharks are threatened by climate change. There is emerging evidence that changes in water temperature and acidity can cause deficiencies in teleost fish cognition in a synergistic manner (Chivers et al. 2014), but far less work has been done in sharks (Pistevos et al. 2017; Rosa et al. 2017). Dixon et al. (2015) found that the ability of smooth dogfish (*M. canis*) to track food odours under future climate scenarios was significantly depleted under high CO_2_ scenarios suggesting that the sensory system of sharks may be impaired. Villa-Pouca et al. (2018) reared juvenile Port Jackson sharks at present day temperatures and in temps 3 °C above present day. Survival of embryos was substantially lower at high temperatures, particularly shortly after hatching. However, those that did survive tended to be more strongly lateralized, that is they developed a strong right turn bias when tested in a detour task. Laterality has been linked to enhanced cognition in a wide variety of animals including fishes (Bisazza and Brown 2011), making it reasonable to hypothesise that if sharks reared at high temperatures are more lateralised, they might also perform better in tests of cognition (Gatto et al. 2019). Villa-Pouca et al. (2019) used a quantity discrimination task to test and compare the learning ability of Port Jackson sharks reared at normal temperature and at + 3 °C. All sharks were trained with the same numerical contrast: 3 dots versus 6 dots. For half of the animals the smaller quantity was reinforced, and for the other half the larger quantity was the positive stimulus. In each trial a different arrangement of the dots was used to avoid identification based on pattern recognition. The hatchlings incubated at elevated temperature, that is the strongly lateralized sharks, performed better in a series of metrics; 33% of the ‘cold’ sharks and 60% of the ‘hot’ sharks were able to learn the task and the hot sharks took half the number of days to learn. These results give a first glimpse that cognition may be impacted by higher temperatures predicted under near-future climate scenarios. Intriguingly, sharks from different parts of the species distribution show variation in their response to climate change with those in the warmer parts of the range showing greater resilience (Gervais et al. 2020).

## Future directions

Despite an upsurge in studies on shark cognition over the last 10–15 years it is still a comparatively unexplored field with most studies centering around a few ‘model’ species, thereby largely neglecting most of the 1200 + elasmobranch species known. Also, most of the research conducted so far has focused on a variety of behavioural topics (far from comprehensive) but in many of these areas follow up studies are either being conducted or are still needed to gain a more holistic understanding of the processes and mechanisms involved as well as the intra-and interspecific breadth of performance. As in other animals, most studies have found large individual variation even within species, with potential cognitive differences existing between sexes, different age cohorts as well as in populations living in different environments. Lastly, while the behaviour is being investigated on several levels, the neural substrates and processes underlying these behaviours are largely unknown. Apart from a few studies on nurse sharks (Graeber and Ebbesson 1972; Graeber et al. 1973, 1978) and bamboo sharks (Schwarze et al. 2013; Fuss et al. 2014a, b, c; Fuss and Schluessel 2018), there have been no studies investigating cognitive behaviour and neural substrates concurrently. It has been suggested that brain size in sharks may correlate with habitat, lifestyle or cognitive capabilities (Bauchot et al. 1976; Northcutt 1977; Yopak et al. 2007; Yopak and Frank 2009; Yopak 2012a, b). Larger brains may be found in pelagic or benthopelagic sharks that live in more complex habitats (such as reefs) and actively prey on others (Yopak 2012a, b). Social species are also likely to possess larger brains, for example those that aggregate and exhibit complex courtship or mating behaviours (Yopak 2012a, b). Several reviews on the brain and specifically on the neuroecology of elasmobranchs have been provided by Ebbesson (1972, 1980), Northcutt (1977, 1978), Collin (2012) and Yopak (2012a, b) but this information has yet to be linked with cognitive performance.

Like other animals, individual sharks have personality which not only dictates their behaviour and responses to stressors (Byrnes and Brown 2016; Finger et al. 2016; Dhellemmes et al. 2020) but likely induces cognitive bias. Nothing is known about how personality shapes cognition in elasmobranchs. Given what we currently know about shark intelligence, it seems reasonable that this should have implications for how humans interact with sharks from a welfare perspective. It is well established that fish have emotions and that particular parts of their brains are associated with emotional learning (e.g. Kittilsen 2013; Broglio et al. 2011; Schwarze et al. 2013). While research on cognitive bias in teleost fishes is still very much in its infancy (Espigares et al. 2022), nothing is known about how emotional states shift perceptions, priorities and ultimately learning styles in sharks and rays.

Teleost fishes are commonly used as models for ecotoxicology in freshwater systems, but much less is known about the impacts in marine systems, least of all on sharks and rays. Data suggest that changing temperatures and pH change fish physiology and behaviour (Munday et al. 2013), sharks may also be impacted in similar ways. Ecotoxicology research shows that common pollutants like zinc can affect the chemosensory systems of a range of organisms interfering with their capacity to detect key amino acids used for identifying predators and prey (Oulton et al. 2014). Recent work on Port Jackson sharks shows that dosing with zinc sulphate reduces their olfactory sensitivity to methionine (Ryan et al. unpublished data) which presumably has implications for cognition across a range of contexts, although the fitness implications have yet to be studied. A fascinating new field is emerging at the intersection between ecotoxicology and aquatic chemical ecology (Gross 2022), which highlights how pollutants interfere with chemical communication systems.

Interestingly, there are also applied uses of our understanding of learning and memory in elasmobranchs outside of understanding their responses to food provisioning as outlined above. Janssen et al. (2017), for example, point out that sharks in captivity can be trained to improve access to vets for health checks or to establish individually tailored feeding regimes. There is also growing interest in the welfare of sharks kept in captivity where environmental enrichment may well have positive impacts on brain development and behaviour as it does in teloeost fishes (Zhang et al. 2021). Finally, a greater understanding of shark cognition is driving the development of solutions to solve human-shark conflicts on our beaches (Egeberg et al. 2019).

## Conclusions

Despite the popular belief that sharks are mindless killing machines, evidence over the last 20 years or so has shown that their cognitive tool box seems to be much like those of other vertebrates. Sharks and rays are intelligent and inquisitive creatures with surprising behavioural flexibility, able to learn using natural and artificial cues. Perhaps this should not come as a surprise since sharks and their relatives have dominated the oceans as top predators for 450 million years and many have surprisingly large brain to body size ratios (Yopak 2012a, b). Like other animals, they are constantly learning about their environment and likely use a mix of public and private information. They also occasionally make mistakes, and this can have consequences for human bathers (Ryan et al. 2021). While elasmobranchs are much maligned, they are also one of the most threatened groups of animals on the planet, with just 25% of species considered safe by the IUCN. We can only hope these remarkable creatures make it through the Anthropocene where they are threatened by over-fishing, climate change, pollution, and habitat degradation (Jorgensen et al. 2022). It’s likely that some of these threats will impact shark cognition in unexpected ways not just by changing the environment in which these animals live but also how they perceive and respond to it.

## Data Availability

Not applicable.
